# P-331. Empowering Black Women in Trusted Community Spaces: Sexual Health and HIV Prevention Initiative Outside of the Doctor’s Office

**DOI:** 10.1093/ofid/ofaf695.550

**Published:** 2026-01-11

**Authors:** Oni Blackstock, Latesha Elopre, Jewel Sawyer, Kelly E Pillinger, Leah Molloy, Laura Simone, Chris Napolitan, Jeffrey D Carter, Melissa Rodriguez, Jenniffer A Meza Jimenez, Shaina Y Vincent

**Affiliations:** Health Justice, New York, NY; University of Alabama of Birmingham, Birmingham, AL; AvitaCare Atlanta, Atlanta, Georgia; PRIME Education, New York, NY; PRIME Education, New York, NY; PRIME Education, LLC, Fort Lauderdale, Florida; PRIME Education, New York, NY; PRIME Education, LLC, Fort Lauderdale, Florida; PRIME Education, New York, NY; PRIME Education, New York, NY; PRIME Education, New York, NY

## Abstract

**Background:**

Black women face a disproportionate burden of HIV diagnoses and unique barriers to sexual health services. This project aimed to enhance their sexual health literacy and linkage to HIV prevention services.

**Table 1.**
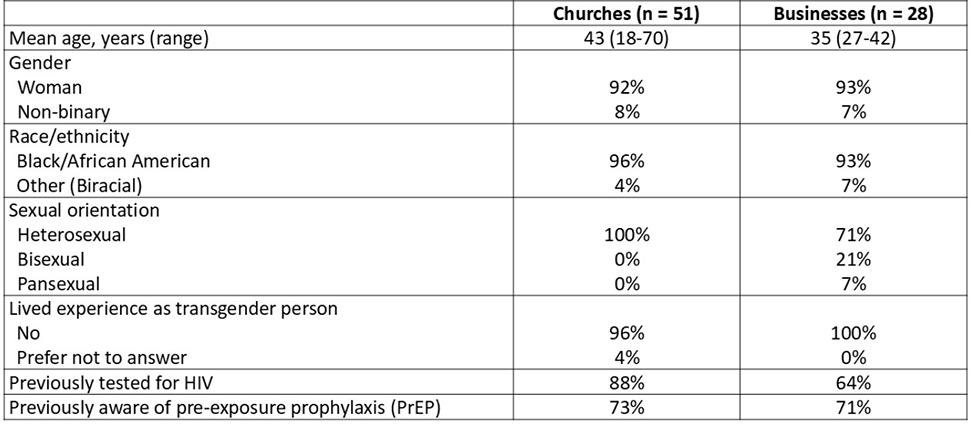
Attendee Demographics from Community Education Sessions Held in Black-Owned Businesses (Hair Salon and Juice Bar) and Churches

**Figure.**
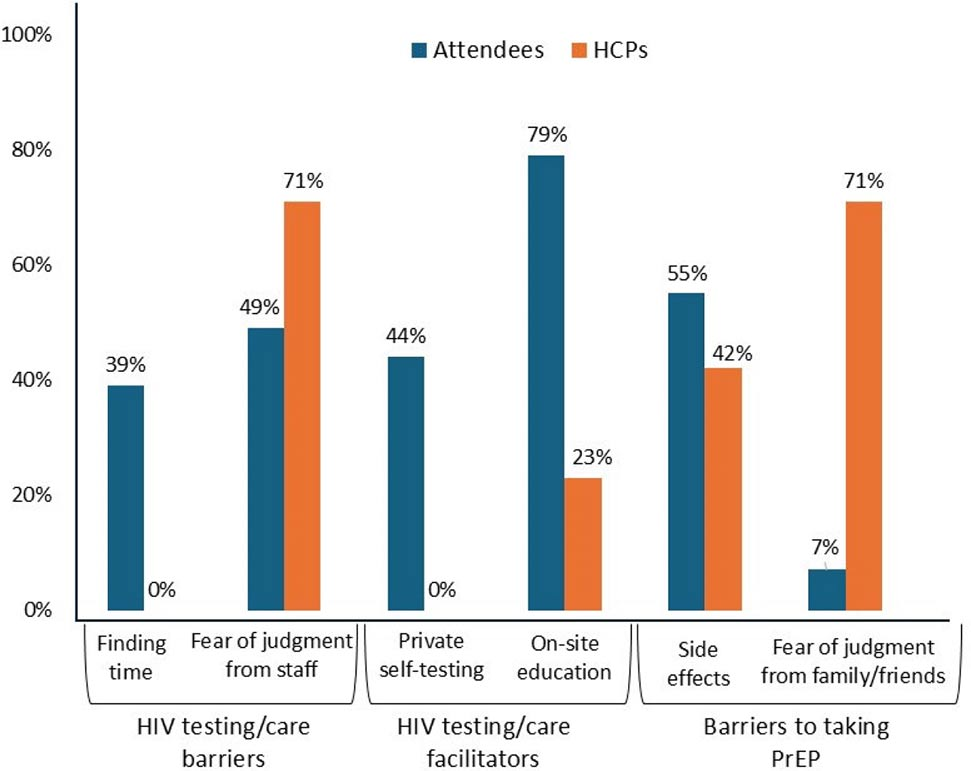
Discordant Barriers and Facilitators to HIV Testing, Care, and Prevention as Perceived by Attendees and Healthcare Professionals (HCPs)

**Methods:**

In 2024, local healthcare professionals (HCPs) led 5 sexual health and HIV prevention sessions in Black-owned businesses (GA, TX) and churches (NY, NC) for cis and transgender Black women. Community leaders also attended to facilitate ongoing community discussions. Attendees and HCPs completed surveys before, after, and longitudinally following each session. Months later, qualitative insights on the program’s impact were gathered from HCPs and leaders.

**Table 2.**
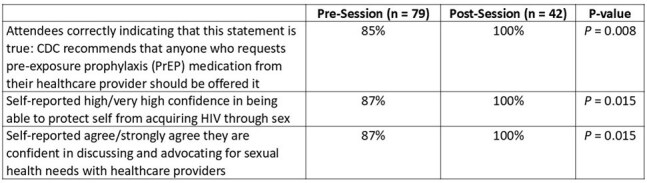
Knowledge and Confidence Regarding HIV Prevention Among Black Women Attendees

**Results:**

A survey of 79 Black women (Table 1) and 24 HCPs revealed contrasting views on HIV testing and prevention barriers and facilitators for Black women (Figure). HCPs perceived fear of judgment from HCPs/clinic staff and social circles as barriers to HIV testing and pre-exposure prophylaxis (PrEP), while attendees cited practical concerns like finding time and potential side effects. For facilitators, attendees favored private self-testing options, whereas HCPs perceived on-site education about the next steps would be favored by Black women. Notably, attendees showed increased knowledge about PrEP eligibility, self-reported confidence in HIV prevention, and advocacy for their sexual health needs with HCPs (Table 2). 3 weeks later, 55 attendees completed follow-up surveys: 31 (56%) reported they had been tested for HIV and 80% discussed HIV/STI prevention with HCPs or scheduled appointments. Within 5 months, 10 participating HCPs reported discussing sexual health with 66 Black women, facilitated HIV testing for 25, and PrEP access for 117. Sustained engagement was observed, with 84% of attendees and 90% of HCPs continuing conversations about sexual health in non-clinical settings. Qualitative follow-up data revealed the importance of trusted, women-centered spaces, dispelling PrEP myths, and empowering Black women to share information within their community.

**Conclusion:**

Culturally tailored, women-centered education in trusted spaces improved HIV prevention knowledge, testing, and PrEP access among Black women, while also identifying barriers and facilitators to sexual health and HIV prevention.

**Disclosures:**

Oni Blackstock, MD, MHS, Gilead Sciences: Speaker and Member of RSP committee|ViiV Healthcare: Advisor/Consultant|ViiV Healthcare: Speaker Latesha Elopre, MD, Gilead Sciences: Advisory Board|Merck: Grant/Research Support|ViiV: Advisory Board

